# Supplementary figures and images for: Estimation of Granger causality through Artificial Neural Networks: applications to physiological systems and chaotic electronic oscillators
Source: PeerJ Comput Sci. 2021 May 18;7:e429. doi: 10.7717/peerj-cs.429 (PMC8157130; doi:10.7717/peerj-cs.429)

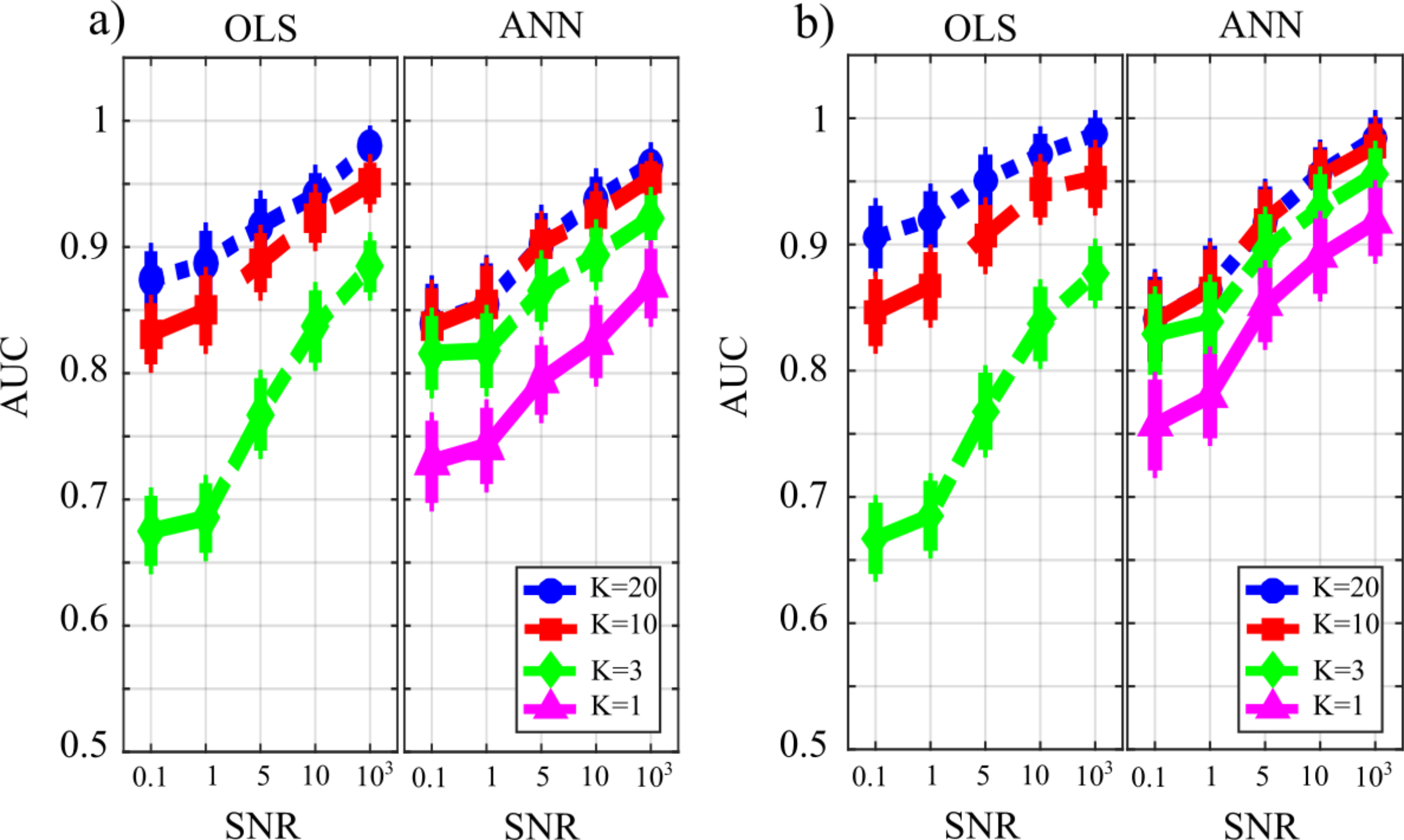

Supplement: Supplemental Information 1 — Plots depict the distribution of AUC expressed as mean value and 95% confidence interval across 100 simulated network as a function of the ration between data samples available and number of parameters to be estimated (K) and of the ratio between signal amplitude and noise amplitude (SNR) for OLS estimation and ANN estimation. Panel a is representative of the AUC computation as described in the main document with the panel b reporting the trends obtained with a quantile based thresholding criteria by using 20 equally-spaced quantiles. [file peerj-cs-07-429-s001.png]
